# The Effect of Lecithins Coupled Decorin Nanoliposomes on Treatment of Carbon Tetrachloride-Induced Liver Fibrosis

**DOI:** 10.1155/2020/8815904

**Published:** 2020-12-13

**Authors:** Guojun Chen, Yiping Zhu, Xiao Liang, Xianfa Wang, Weihua Yu, Junping Guo, Linghua Zhu, Rui Ma

**Affiliations:** ^1^Department of General Surgery, Sir Run Run Shaw Hospital, College of Medicine, Zhejiang University, Hangzhou, Zhejiang 310016, China; ^2^Zhejiang University City College, Hangzhou 310015, China; ^3^Department of Surgery, Zhejiang University Hospital, Zhejiang University, Hangzhou, Zhejiang 310027, China

## Abstract

This study aimed to investigate the effect of bile duct-targeting lecithins- (PC-) coupled decorin (DCN) (PC-DCN) nanoliposomes against liver fibrosis in vitro and in vivo. We prepared PC-DCN nanoliposomes by using rat astrocytes, HSC-T6, to verify the antifibrosis effect of PC-DCN in vitro. First, we established a rat model of carbon tetrachloride-induced fibrosis. PC-DCN nanoliposomes were then injected into fibrotic rats via the portal vein or bile duct. The EdU assay was performed to analyze cell proliferation. Immunofluorescence staining was used to detect *α*-smooth muscle actin (*α*-SMA) expression. Western blot was performed to examine the expression of *α*-SMA, collagen type I alpha 1 (COL1A1), and transforming growth factor-*β* (TGF-*β*) protein. The levels of aspartate transaminase (AST), alanine transaminase (ALT), and total bilirubin (TBIL) were examined by enzyme-linked immunosorbent assay (ELISA) analysis. Hematoxylin and eosin (H&E) staining and Masson trichrome staining were used to determine liver tissue lesions and liver fibrosis. Compared with TGF-*β* group, PC-DCN treatment could significantly reduce cell proliferation. Western blot analysis indicated that the expression of *α*-SMA, COL1A1, and TGF-*β* was downregulated after treatment with PC-DCN in vitro and in vivo. Immunofluorescence staining confirmed that *α*-SMA expression was reduced by PC-DCN. Furthermore, H&E staining and Masson trichrome staining showed that the administration of PC-DCN nanoliposomes via the bile duct could reduce the extent of liver fibrosis. PCR analysis showed that PC-DCN administration could reduce proinflammatory cytokines IL-6, TNF-*α*, and IL-1*β* expression via the bile duct. The administration of PC-DCN nanoliposomes also significantly downregulated liver function indicators ALT, AST, and TBIL. The results of our study indicated that PC-DCN could effectively reduce the extent of liver fibrosis.

## 1. Introduction

Liver fibrosis has become one of the common diseases threatening human health and largely affects the quality of life of the affected patients [1]. It progresses to irreversible liver cirrhosis, even liver cancer, if not diagnosed in a timely manner or if effective treatments are lacking [2]. Liver fibrosis is mainly caused by the excessive deposition of extracellular matrix (ECM) [3]. In chronic liver injury, the excessive deposition of ECM, especially collagen fibrils, leads to the breakdown of the balance between synthesis and degradation, resulting in the loss of regeneration ability [4]. Although great progress has been made in research on liver fibrosis, the complexity of its pathogenesis, the most critical problem is that there is still a lack of an ideal and effective treatment to reverse liver fibrosis. Therefore, it is imperative to explore the mechanism underlying the development of liver fibrosis and search for effective treatment methods.

Current studies suggest that liver fibrosis is characterized by activation of hepatic stellate cells (HSCs) [1]. HSC activation is the central link in the development of liver fibrosis, and all types of fibrosis pathogenic factors jointly initiate the process of liver fibrosis. HSCs are closely associated with the development and treatment of liver fibrosis and have become a hotspot in the study of liver fibrosis. TGF-*β*1, the primary subtype, one of the most important proliver fibrosis peptide growth factors, plays an important role in the process of liver fibrosis [5]. In addition to the promotion the process of a profibrogenic, TGF-*β* is also used as a strong promoter of cancer [6, 7]. Therefore, inhibition of TGF-*β*1 activation in HSCs is an important research direction for developing interventions for the management of liver fibrosis. Decorin (DCN) is an ECM component; it is rich in leucine and composed of a core protein and a glycosaminoglycan chain [8]. DCN exerts antifibrosis effects, which are mainly attributable to its core protein. In a previous study, we found that DCN could relieve liver fibrosis following chronic carbon tetrachloride- (CCl_4_-) induced liver injury [9]. The existing research on fibrosis is mostly focused on nontargeted interventions, which cannot specifically affect the fibrosis and cause a certain degree of damage to the normal cells around the lesion, leading to complications [2, 10]. To overcome these obstacles and improve stability, we have designed a nanocarrier which was mainly composed of lecithin to efficiently target liver disease. The preparation method of liposomal particles is simple, easy to carry out surface modification, has high biocompatibility, and is a great candidate materials. Sato et al. proposed a new research direction for HSC liposomes, whereby vitamin A-coupled liposomes were used to encapsulate siRNAgp46 to target HSCs and satisfactory results were achieved [11]. Accumulating evidence has shown that vitamin A-coupled liposomes are effective in treating all types of fibrosis, such as skin, pulmonary, and pancreatic fibrosis [12–14].

In the current study, we developed a lecithin-based nanoliposome coupled to DCN and investigated the antifibrosis effect of PC-DCN through bile duct administration of the nanoliposomes in vitro and in vivo.

## 2. Methods

### 2.1. Preparation of Liposomes

The DCN plasmid was constructed with lecithin as the main lipid and cholesterol and octadecylamine as additional agents to adjust the fluidity of the bilayer. Using an analytical balance, 0.1 g of lecithin, 0.01 g of cholesterol, and 0.004 g of octadecylamine were accurately weighed and added into a 5 mL beaker, to which was then added 10 mL of chloroform to fully dissolve the compounds at room temperature to obtain a light yellow clear solution. Transferred the solution into a 100-mL round-bottom flask. Finally, chloroform was completely removed by evaporation under reduced pressure in a water bath maintained at 37°C to obtain a lipid membrane. (2) Another 33 *μ*g of the plasmid was weighed accurately and dissolved in 5 mL of pure water for use. (3) The solution (2) was then added to the (1) 100-mL round-bottom flask, which was then placed on a magnetic stirrer. The contents of the flask were stirred and hydrated at room temperature for 30 min to obtain the plasmid-encapsulated lipid body suspension. (4) Ultrasonic treatment: the plasmid liposome suspension was fully ultrasonicated with a cell crusher at 60 Hz twice for 30 s each time to obtain a uniform transparent solution.

### 2.2. Establishment of the Rat Model of Liver Fibrosis and the Administration of PC-DCN

SD rats (male, 4-6 weeks, 180-220 g) were purchased from Charles River (Beijing, China). Liver fibrosis was induced in rats by intraperitoneal injection of 50% (*v*/*v*) CCl_4_ diluted in olive oil (1 mL/kg body weight) three times a week for 8 weeks. Significant liver fibrosis was confirmed by pathologic analysis in all animals after treatment. The model animals were randomly divided into three groups (*n* = 6): CCl_4_ group (model group; treated with CCl_4_ alone), treatment 1 (portal vein injection of 30 *μ*g/500 *μ*l PC-DCN), and treatment 2 (bile duct administration of 30 *μ*g/500 *μ*l PC-DCN). The control group was treated with commensurable vehicle (olive oil only). In the portal vein administration group, the abdominal cavity was incised, and PC-DCN was injected into the portal vein. Then we sutured the abdominal cavity andobserved the animal for a week. In the bile duct administration group,we incised the abdominal cavity, and injected PC-DCN into the common bile duct injection, used 32g puncture needle to puncture the common bile duct, then pull out the inner core, used 5-0 silk thread to fix puncture needle, continuous injected PC-DCN at a rate of 0.2 ml/min, ligated the common bile duct between the injection point and the liver for 20 minutes to prevent direct reflux after injection completed, pulled out the puncture needle; closed the abdomen and observed for a week. The animals were killed 2 weeks later.

### 2.3. Cell Culture

HSC-T6 cells were purchased from Procell Life Science & Technology Co., Ltd. (Wuhan, China; Cat NO.: CL-0116) and grown in DMEM + 10% FBS + 1% P/S at 37°C under incubation (95% air, 5% CO_2_).

### 2.4. Western Blot Analysis

The cells were lysed with RIPA buffer (Beyotime Biotechnology), and protein concentrations were determined using the BCA protein assay. Forty micrograms of the sample were run using 10% SDS-PAGE, and the sample was then transferred to a PVDF membrane. The membrane was blocked with 5% skin milk powder for 2 h at 37°C; washed thrice with TBS-T; and incubated with anti-*α*-SMA (Abcam; ab7817; 1 : 1000), anti-COL1A1 (Abcam; ab34710; 1 : 1000), anti-TGF-*β* (Proteintech; 18978; 1 : 1000), and anti-DCN antibodies (Origene; TA327131; 1 : 1000) overnight at 4°C, washed with TBS-T for three times under the shaker with slow speed, incubated with corresponding secondary antibodies (Santa Cruz, USA; 1 : 2000) at 37°C for 1 h under the shaker with slow speed. The membrane with the protein bands were detected using a chemiluminescence system (ECL; Beyotime Biotechnology, Shanghai, China).

### 2.5. Cell Proliferation

Cell proliferation was determined by EdU staining according to the manufacturer's (Abcam, USA) protocols.

### 2.6. Liver Function Analysis

Blood samples were collected from the abdominal aorta of the liver transplant models, and serum levels of liver function markers, aspartate aminotransferase (AST), alanine aminotransferase (ALT), and total bilirubin (TBIL) (Nangjing Jiancheng Bioengineering Institute, Nanjing, China) were detected using an automated chemistry analyzer (Beckman, CA, USA).

### 2.7. Oil Red O Staining

Oil Red O staining was performed to determine the steatosis of tissues and organs owing to abnormal lipid deposition.

### 2.8. Immunofluorescence Analysis

After deparaffinized the paraffin sections and dehydrated with gradient alcohol, performed antigen retrieval, and then rinsed with 0.01 M PBST for 5 min × 3/time; blocked with 5%BSA in 37°C wet box for 30 min. Then, incubated with primary anti-*α*-SAM (1 : 100; Abcam) at 4°C overnight, washed with PBS, and incubated with species-matched secondary antibodies (1 : 200; Beyotime Biotechnology) for 30 min at room temperature. Finally, the samples were incubated with DAPI (Sigma) for 5 min, washed twice in PBS, and examined by a fluorescence microscope (Olympus, Tokyo, Japan) to produce a merged image.

### 2.9. Histological Evaluation

Carry out paraffin embedding, formaldehyde fixation, and sectioning of liver tissue. Medium lipid status, pathological findings, and collagen accumulation were assessed using hematoxylin and eosin (H&E) staining, and Masson staining, respectively. Quantification with the Image-Pro Plus 6.0 software was performed by calculating the ratio of connective tissue to the total liver area.

### 2.10. Immunocytochemistry

Immunohistochemical staining was performed on paraffin-embedded tissue sections (5 mm) to determine *α*-SAM and TGF-*β* expression. The slides were incubated with anti-*α*-SAM and anti-TGF-*β* antibodies (1 : 500, Abcam) overnight at 4°C. Washing with PBS for 3 times, dried the slices with absorbent paper and incubated with a horseradish peroxidase (HRP) secondary antibody (Cell Signaling Technology) at 37°C for 30 min washing again, added the diaminobenzidine (DAB) (Abcam; ab64238) to obverse the staining under a light microscope, later counterstained with hematoxylin for 30 s, dehydrated, mounted, and observed under a light microscope (Olympus, Tokyo, Japan).

### 2.11. Real-Time PCR

Trizol Reagent (Invitrogen, Carlsbad, CA, USA) was used to extract total mRNA from samples. cDNA was then synthesized from 2 *μ*g of total RNA using MMLV reverse transcriptase (Promega, WI, USA). Real-time PCR was performed in triplicate using a SYBR PrimeScript RT-PCR Kit to measure TNF-*α*, IL-6, and IL-1*β* mRNA expression. (Takara, China). GAPDH mRNA acts as a loading control. The data were collected and quantitatively analyzed using a Mx4000 system (Stratagene, La Jolla, CA).

### 2.12. Statistical Analysis

The experimental data were expressed as mean ± SD values and analyzed by Student's *t*-test or one-way analysis of variance with a post hoc test. Statistical analysis was determined using the GraphPad Prism 8. The values of *P* < 0.05 were considered statistically significant.

## 3. Results

### 3.1. Preparation of the PC-DCN Liposome

**Figure 1:**
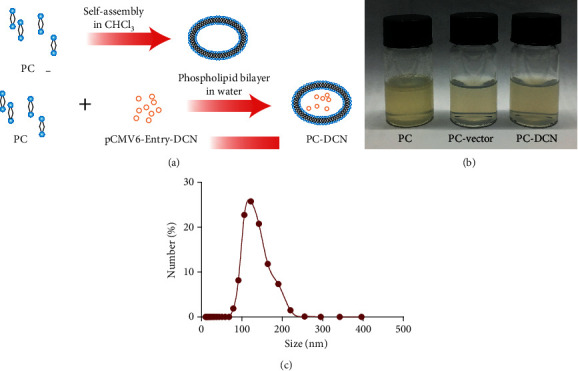
Preparation of the PC-DCN liposome, (a) Schematic illustration of the preparation of PC-DCN liposomes. (b) Characterization of liposomes by schematic diagram. (c) The average particle size of PC-DCN was represented by a histogram.

To investigate the antihepatic fibrosis effects of DCN, we prepared the PC-DCN liposome; a schematic diagram of the preparation protocol is shown in Figure 1(a). Next, we observed the characteristics and particle size of the liposomes. The liposomes were assessed for 24 h after preparation, during which we found that while PC was soluble in chloroform, PC-vector and PC-DCN were soluble in water, indicating that the ratio of coupled liposome is relatively good (Figure 1(b)). The results of dynamic light scattering analysis revealed that the average hydrodynamic diameter of PC-DCN is 118.75 nm (Figure 1(c)).

### 3.2. PC-DCN Reduced the Elevated TGF-*β*-Induced Cell Proliferation of HSC-T6 Cells by Inhibiting Fibrosis-Related Protein Expression

**Figure 2:**
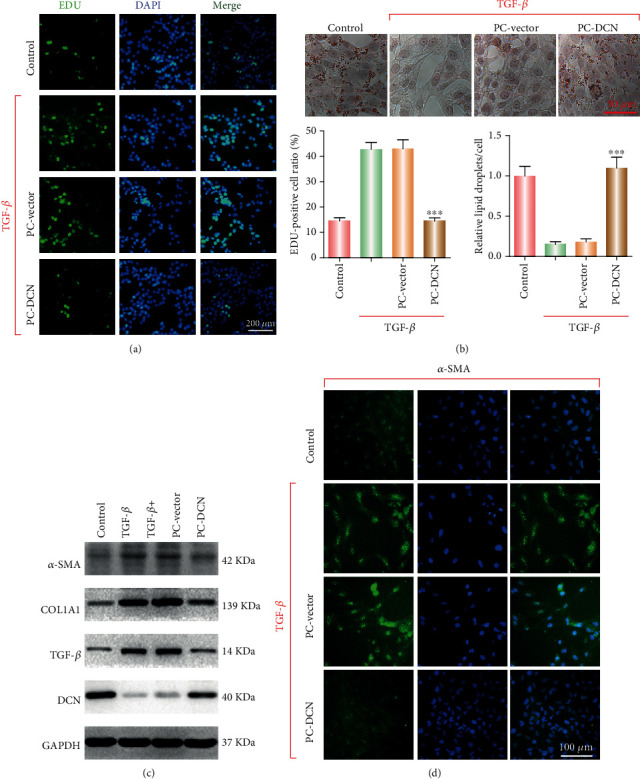
PC-DCN reduced cell proliferation in HSC-T6 cells by inhibiting fibrosis-related protein expression. (a) EdU assay for determining cell proliferation following treatment with PC-DCN. ^∗∗∗^*P* < 0.001 vs. control. (b) Oil Red O staining was performed to analyze the lipid status in different groups. (c) Western blotting was used to detect the expression of fibrosis-related proteins (*α*-SMA, COL1A1), TGF-*β*, and DCN after treatment with or without PC-DCN. ^∗∗∗^*P* < 0.001 vs. control. (d) The *α*-SMA expression after treatment with PC-DCN was examined by immunofluorescence analysis.

To further study the antifibrotic effect of PC-DCN liposomes, we used rat HSC-T6 astrocytes stimulated with TGF-*β* to yield active HSC-T6 cells. TGF-*β*1, a powerful fibrillary cytokine, stimulates collagen type I expression in primary HSCs [15]. As shown in Figure 2, EdU analysis indicated that PC-DCN could significantly inhibit the TGF-*β*-induced proliferation of HSC-T6 cells (Figure 2(a)). Oil Red O staining revealed that PC-DCN treatment could significantly reduce the number of TGF-*β*-induced red lipid droplets in HSC-T6 cells, indicating that PC-DCN could inhibit the activation of HSC-T6 cells following TGF-*β* stimulation (Figure 2(b)). Western blot detected expression changes in fibrosis indicators, indicating that in comparison with the TGF-*β* group, *α*-SMA, COL1A1, and TGF-*β* protein expression was significantly downregulated following treatment with PC-DCN (Figure 2(c)). Immunofluorescence analysis was performed to detect the expression of *α*-SMA in fibrotic liver, the results showed that the expression of *α*-SMA fluorescence after PC-DCN treatment was downregulated in comparison with that in the TGF-*β* group (Figure 2(d)). These results indicated that PC-DNC could inhibit the increase of TGF-*β*-induced cell proliferation in HSC-T6 cells by inhibiting the expression of fibrosis-related index proteins.

### 3.3. The Antifibrotic Effects of PC-DCN In Vivo

**Figure 3:**
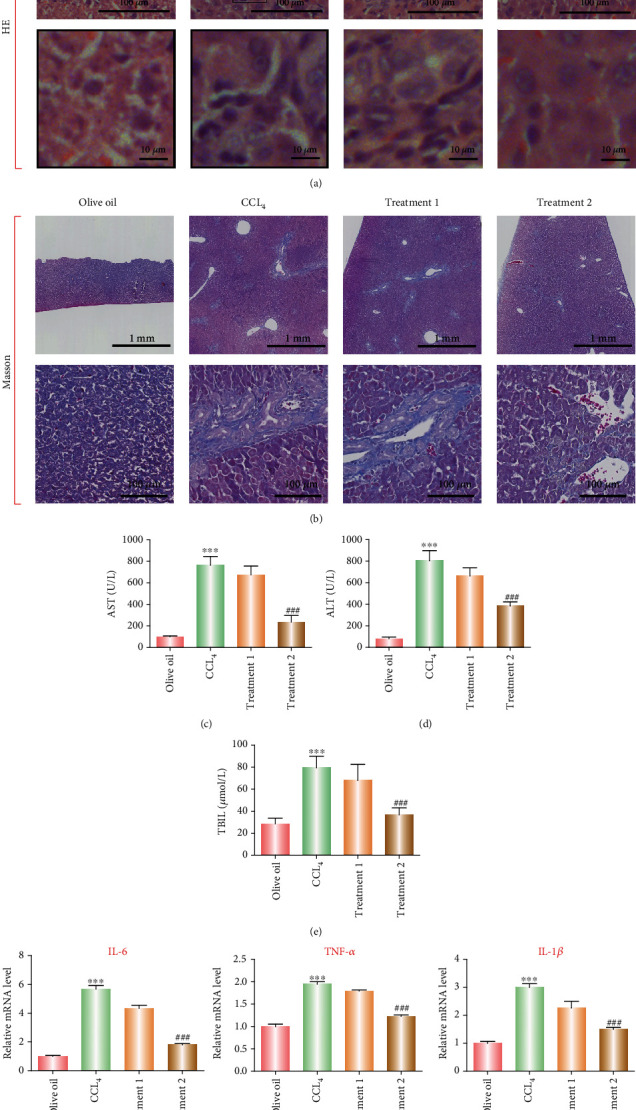
Antifibrotic effect of PC-DCN in vivo. (a) The histopathology of liver tissue in different treatment groups (control, CCl_4_) stained using H&E. (b) Masson staining showed the changes in liver collagen fibers. (c–e) The levels of ASL, ALT, and TBIL under different treatment regimens were detected by ELISA. ^∗∗∗^*P* < 0.001 vs. control; ^###^*P* < 0.001 vs. CCl_4_. (f) Real-time PCR confirmed proinflammatory cytokines IL-6, TNF-*α*, and IL-1*β* expression after treatment with PC-DCN.

We firstly established a rat fibrosis model to investigate the antifibrotic effect of PC-DCN in vivo. PC-DCN liposomes were injected into the fibrotic rats via the portal vein or bile duct after induction with CCl_4_. Next, we observed the condition of the fibrotic liver following treatment with PC-DCN liposomes and PC blank liposomes. HE staining results showed that the normal liver tissue structure was clear and complete; CCl_4_ induction resulted in a large amount of neutrophil infiltration. In comparison with the CCl_4_ group, PC-DCN bile duct administration reduced the extent of liver fibrosis, but portal vein administration did not reduce significantly the extent of liver fibrosis (Figure 3(a)). Masson trichrome staining showed a significant reduction in liver collagen fibers and muscle fibers after bile duct administration in comparison with the CCl_4_ group **(**Figure 3(b)**)**. In addition, we also tested the recovery of liver function indexes such as serum ALT, AST, and TBIL levels in rats and found that in comparison with mice showing CCl_4_-induced liver fibrosis; the expression of ALT, AST, and TBIL was significantly downregulated after PC-DCN administration via the bile duct (Figures 3(c)–3(e)). Furthermore, we found that proinflammatory cytokines IL-6, TNF-*α*, and IL-1*β* was significantly downregulated after PC-DCN administration via the bile duct (Figure 3(f)), indicating that PC-DCN could reduce liver inflammation.

**Figure 4:**
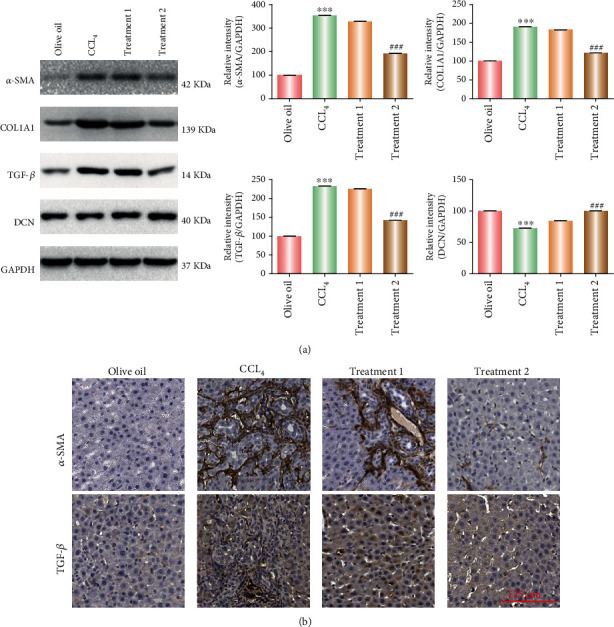
(a) Western Blot analysis of *α*-SMA, COL1A1, TGF-*β*, and DCN protein expression following PC-DCN intravenous or bile duct administration under CCl_4_ induction in mice. ^∗∗∗^*P* < 0.001 vs. control; ^###^*P* < 0.001 vs. CCl_4_. (b) H&E staining showing the expression of *α*-SMA and TGF-*β* following PC-DCN intravenous or bile duct administration under CCl_4_ induction in mice. ^∗∗∗^*P* < 0.001 vs. control; ^###^*P* < 0.001 vs. CCl_4_.

**Figure 5:**
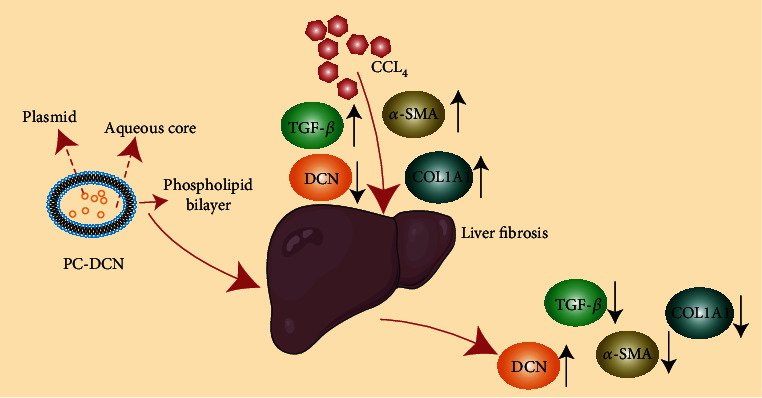
Schematic illustration of PC-DCN inhibition of CCl_4_-induced liver fibrosis.

Next, western blotting was performed to detect the changes of fibrosis indicators, and the findings showed that in comparison with the corresponding levels in CCl_4_-induced group, the expression levels of *α*-SMA, COL1A1, and TGF-*β* proteins were all downregulated after bile duct administration (Treatment 2), while the changes in these proteins are not particularly obvious following the portal vein administration (Treatment 1) (Figure 4(a)). Immunofluorescence analysis also confirmed consistent results for the expression of *α*-SMA and TGF-*β*. These results suggest that PC-DCN treatment via bile duct administration could inhibit CCl_4_-induced liver fibrosis. The research schematic is shown in Figure 5.

## 4. Discussion

Liver fibrosis is a complex disease, and its main feature is the excessive accumulation of ECM proteins which contain collagen. In the normal liver, HSCs are in a state of quiescence and nonproliferating. However, HSCs are activated and then differentiate into myofibroblasts after liver injury or culture in vitro, and the myofibroblasts have the characteristics of cell proliferative, contraction, inflammation, and chemotaxis by enhanced extracellular matrix (ECM) production [16]. The current research results revealed for the first time the protective effects of PC-DCN on liver fibrosis in vitro and in vivo.

Decorin, a natural antifibrotic molecule, can bind with high affinity to TGF-*β* and prevent its interaction with profibrotic receptors. TGF-*β* is generally considered to be the most effective fibrous cytokine, released in latent form by several cell populations in the liver [17]. Inflammation contributes to the fibrosis and scar formation. Decorin has a significant anti-inflammatory effect in fibrosis-related inflammatory diseases. DCN (KO mice) gene deletion is a proinflammatory (and profibrotic) sign, whereas treatment with recombinant DCN or DCN gene has shown anti-inflammatory effects [18, 19]. Decorin also reduces liver fibrosis after CCl_4_-induced liver injury [9]. However, most of the existing studies on fibrosis are nontargeted interventions, and some problems need to be solved urgently. It cannot specifically act on fibrous lesions, and it damages normal cells around the lesions to a certain extent, leading to complications [10, 20]. Targeted drug delivery has a major impact on the therapeutic effect. The most prominent feature of targeted therapy is that it delivers the drugs to the target area to the maximum extent. The specificity of targeted therapy is high, the local effect is strong, and the systemic toxicity is low. At the same time, targeted drug delivery can improve the efficacy of drugs and reduce toxic reactions. As a new drug delivery method, it has been valued by domestic and foreign counterparts. The existing research on the use of hepatic stellate cells to target antihepatic fibrosis mostly focuses on the antifibrotic effects of reducing HSC activation and collagen deposition in fibrous foci [11, 21]. In our study, we synthesized PC-DCN nanoliposomes and analyzed the characteristics and particle sizes of the liposomes to verify their stability. Then, we found that PC-DCN could inhibit cell proliferation and the expression of *α*-SAM after treatment with TGF-*β* in HSC-T6 cells. However, there is currently no in vivo experimental study that directly inhibits liver fibrosis, and research on the effects of DCN against liver fibrosis is limited.

Conventional intravenous injections of nanodrugs have been reported to often present with acute manifestations such as fibrous focal hepatic sinus endothelial blockage, resulting in portal hypertension [22]. To solve the problem of blocked hepatic sinusoidal endothelium, a novel drug delivery method biliary retrograde delivery has emerged. Biliary tract delivery offers the following advantages: it can reduce the contact of the nanodrug with Kupffer cells of the liver and significantly improve gene expression, avoid the liver microvascular system to the greatest extent, and reduce drug loss when reaching the liver target location [23, 24]. Therefore, biliary tract delivery of targeted drugs has good applicability. In the present study, we established a rat model of CCl_4_-induced liver fibrosis and compared the effects of the two administration methods on liver fibrosis therapy with PC-DCN. In the portal vein administration group, the abdominal cavity was incised, PC-DCN were injected into the portal vein, and then the abdominal cavity was sutured. In the bile duct administration group, the abdominal cavity was cut, the common bile duct was punctured, and the PC-DCN were continuously injected at a rate of 0.2 mL/min. Next, HE staining showed that CCl_4_ induction resulted in a large amount of neutrophil infiltration, while PC-DCN bile duct administration could reduce the extent of liver fibrosis and portal vein administration did not significantly change it. Furthermore, PC-DCN administration could reduce proinflammatory cytokines IL-6, TNF-*α*, and IL-1*β* expression via the bile duct, revealing that PC-DCN could reduce inflammation. PC-DCN bile duct administration also could significantly reduce liver collagen fibers and muscle fibers: ALT, AST, TBIL, *α*-SMA, and TGF-*β* expression, while the findings obtained with the other mode of portal vein administration were not significantly different from those obtained in the CCl_4_ injury group. Our study used HSCs to target DCN gene nanoliposomes for the first time to explore the effect and mechanism of DCN intervention in liver fibrosis. However, we need to screen more effective molecules for liver fiber that bind to PCN. Next, it is necessary for us to realize clinical transformation and we also need to strengthen the role of PC-DCN in clinical transformation. We hope we will early realize cross-combine the materials innovation and medical research results to provide a more effective method for future clinical antifibrosis treatment.

## 5. Conclusion

The results of the current study showed the molecular mechanism of PC-DCN nanoliposomes targeting HSCs in biliary transport against CCl_4_-induced hepatic fibrosis in rats. These results indicate the possibility of a new approach for the diagnosis and treatment of liver fibrosis.

## Data Availability

We declare that all data supporting the conclusions of the study.
